# Supplementary material for: Development of a computer-aided design software for the quantitative evaluation of aesthetic damage
Source: PLoS One. 2019 Dec 18;14(12):e0226322. doi: 10.1371/journal.pone.0226322 (PMC6919621; doi:10.1371/journal.pone.0226322)
Supplement: S1 Code site — 1. (ZIP) [file pone.0226322.s001.zip › [EESC_jr][Projeto]Relatorio_07_21/vendors/select2/docs/_includes/options-old/setting-default-options.html]

# Setting default options

In some cases, you need to set the default options for all instances of
Select2 in your web application. This is especially useful when you are
migrating from past versions of Select2, or you are using non-standard
options like custom AMD builds. Select2 exposes the
default options through `$.fn.select2.defaults`, which allows
you to set them globally.

When setting options globally, any past defaults that have been set will
be overriden. Default options are only used when an option is requested
that has not been set during initialization.

**You can set default options** by calling
`$.fn.select2.defaults.set("key", "value")`. The key that is
set should take the same format as keys set using
HTML `data-*` attributes which
means that two dashes (`--`) will be replaced by a level of
nesting, and a single dash (`-`) will convert it to a camelCase
string.

```
$.fn.select2.defaults.set("theme", "classic");
```

**You can reset the default options** by calling
`$.fn.select2.defaults.reset()`.
